# Roles of miR-20a-5p in breast cancer based on the clinical and multi-omic (CAMO) cohort and in vitro studies

**DOI:** 10.1038/s41598-024-75557-0

**Published:** 2024-10-23

**Authors:** Eline Sol Tylden, André Berli Delgado, Marko Lukic, Line Moi, Lill-Tove Rasmussen Busund, Mona Irene Pedersen, Ana Paola Lombardi, Karina Standahl Olsen

**Affiliations:** 1 Department of Medical Biology, Faculty of Health Sciences, UiT The Arctic University of Norway, Tromso, Norway; 2 Department of Community Medicine, Faculty of Health Sciences, UiT The Arctic University of Norway, Tromso, Norway; 3 Department of Clinical Pathology, University Hospital of North Norway, Tromso, Norway; 4 Department of Clinical Medicine, Faculty of Health Sciences, UiT The Arctic University of Norway, Tromso, Norway

**Keywords:** Breast cancer, miR-20a-5p, microRNA, miRNA, Biomarker, Epidemiology, Tissue microarray, In situ hybridization, Translational research, Breast cancer, Biomarkers

## Abstract

MicroRNAs are involved in breast cancer development and progression, holding potential as biomarkers and therapeutic targets or tools. The roles of miR-20a-5p, a member of the oncogenic miR-17-92 cluster, remain poorly understood in the context of breast cancer. In this study, we elucidate the role of miR-20a-5p in breast cancer by examining its associations with breast cancer risk factors and clinicopathological features, and its functional roles in vitro*.* Tissue microarrays from 313 CAMO cohort breast cancer surgical specimens were constructed, in situ hybridization was performed and miR-20a-5p expression was semiquantitatively scored in tumor stromal fibroblasts, and in the cytoplasm and nuclei of cancer cells. In vitro analysis of the effect of miR-20a-5p transfection on proliferation, migration and invasion was performed in three breast cancer cell lines. High stromal miR-20a-5p was associated with higher Ki67 expression, and higher odds of relapse, compared to low expression. Compared to postmenopausal women, women who were premenopausal at diagnosis had higher odds of high stromal and cytoplasmic miR-20a-5p expression. Cytoplasmic miR-20a-5p was significantly associated with tumor grade. In tumors with high cytoplasmic miR-20a-5p expression compared to low expression, there was a tendency towards having a basal-like subtype and high Ki67. In contrast, high nuclear miR-20a-5p in cancer cells was associated with smaller tumor size and lower odds of lymph node metastasis, compared to low nuclear expression. Transfection with miR-20a-5p in breast cancer cell lines led to increased migration and invasion in vitro*.* While the majority of our results point towards an oncogenic role, some of our findings indicate that the associations of miR-20a-5p with breast cancer related risk factors and outcomes may vary based on tissue- and subcellular location. Larger studies are needed to validate our findings and further investigate the clinical utility of miR-20a-5p.

## Introduction

Breast cancer is now the most common form of cancer worldwide and the leading cause of cancer-related death in women^1^. Identification of biomarkers and new therapeutic targets for early detection, risk stratification and treatment is crucial to reducing breast cancer mortality. The complex systems underlying breast cancer development and progression call for a systems epidemiology approach that integrates clinical, molecular, and multi-omics data, to develop more tailored prevention and treatment strategies through precision medicine^2–4^.

MicroRNAs (miRNAs) are small, endogenous, non-coding RNA (ncRNA) molecules that post-transcriptionally regulate gene expression^5,6^. Depending on their target genes, miRNAs can function as either oncogenes or tumor suppressors to influence various hallmarks of cancer, such as the ability to evade growth suppressors, sustain proliferative signaling, resist cell death, activate migration and metastasis, or induce angiogenesis^7^. MiRNAs are frequently dysregulated in human cancers, and abnormal expression levels of miRNAs can be detected in solid tumors or body fluids, making them potential biomarkers for diagnosis, prognosis and prediction of treatment response^8,9^. Furthermore, miRNAs hold promise as therapeutic targets or tools^7,8^.

A candidate miRNA in this context is miR-20a-5p, a member of the miR-17-92 cluster. This cluster has a dual role in cancer, its members having demonstrated both tumor promoter and tumor suppressor functions in several cancer types^10^. Previous studies have demonstrated the dysregulation of miR-20a-5p in breast cancer, particularly triple negative breast cancer (TNBC). MiR-20a-5p has shown significantly higher expression levels in breast cancer tissue compared to normal breast tissue^11^. Significantly higher expression levels have also been found in TNBC tissue compared to normal breast tissue and non-TNBC tissue^12^, and in TNBC cells compared to HER2-positive^13^ and luminal A^14^ breast cancer cells. Further, high miR-20a-5p expression levels have been found in the TNBC cell line MDA-MB-231 and in the exosomes derived from this cell line^15^.

MiR-20a-5p has been linked to growth and proliferation of breast cancer cells. Bai et al. found that miR-20a-5p overexpression promoted cell growth and proliferation both in vitro and in vivo^16^, while Zhao et al. found the opposite effect in vitro^17^. Similarly, two studies demonstrated that miR-20a-5p overexpression led to increased migration and invasion of TNBC cells in vitro^15,16^, while another study found that the migrative and invasive capabilities were impaired^17^. Further, in vitro studies have suggested a promoting effect of miR-20a-5p on apoptosis^17^ and angiogenesis^18^.

Recent studies have suggested miR-20a-5p as a putative prognostic or predictive biomarker. A panel of eight miRNAs, including miR-20a-5p, was identified as a signature associated with tumor recurrence and decreased survival in TNBC patients^12^. In two independent patient cohorts of metastatic breast cancer patients, Rinnerthaler et al. found that low miR-20a-5p expression in breast cancer tissue predicted a greater benefit from bevacizumab-containing therapy, being significantly associated with longer progression-free and overall survival^19^.

Although previous studies suggest that miR-20a-5p exerts a role in breast cancer development and progression, substantial discrepancies among research findings warrant further investigation into the functional roles and biomarker potential of miR-20a-5p in breast cancer.

Research on the relationship between miR-20a-5p and established breast cancer risk factors, such as lifestyle and reproductive factors, is currently limited. Adopting the systems epidemiology perspective, investigating this relationship can help us understand the effect of these factors on gene expression and their contribution to the initiation and progression of breast cancer.

The emerging understanding that miRNAs may possess both cytoplasmic and nuclear function^20–22^, as well as their involvement in the tumor microenvironment^23^, highlights the importance of visualizing their tissue- and subcellular localization. However, none of the most commonly used techniques for miRNA quantification provide information on miRNA localization within the tissue or cell^24^.

In this study, we evaluated the expression profile of miR-20a-5p in 313 surgical specimens from breast cancer patients within the Clinical and Multi-omic (CAMO) cohort, which is part of the Norwegian Women and Cancer (NOWAC) cohort. The overall aim was to elucidate the role of miR-20a-5p in breast cancer and enhance our understanding of how miR-20a-5p expression is associated with breast cancer biology and its potential role as a biomarker for prognosis or targeted therapy. Specifically, three objectives were defined: (a) to assess the spatial and subcellular expression profile of miR-20a-5p in breast cancer tissues, which has not been previously addressed in the literature, (b) to quantify the association of miR-20a-5p expression with patient and tumor characteristics, including demographics, molecular subtypes, and clinicopathological features, and (c) to investigate the effects of miR-20a-5p on key aspects of breast cancer cell behavior, including proliferation, migration, and invasion.

To our knowledge, this is the first study to comprehensively evaluate the expression level of miR-20a-5p in various tissue- and subcellular compartments, explore its associations with breast cancer risk factors and clinicopathological features, and examine its functions in vitro*.*

## Materials and methods

### Study population

Our study includes a subset of participants from the CAMO cohort, described in detail elsewhere^25^. Nested within the NOWAC cohort^26^, the CAMO cohort consists of 388 women diagnosed with breast cancer in North Norway before 2013. For these women, we have detailed information on demographic, anthropometric, lifestyle, reproductive and clinicopathological parameters. The data was retrieved from questionnaires, medical records, national registries and histopathological analyses of tumor tissue.

**Fig. 1:**
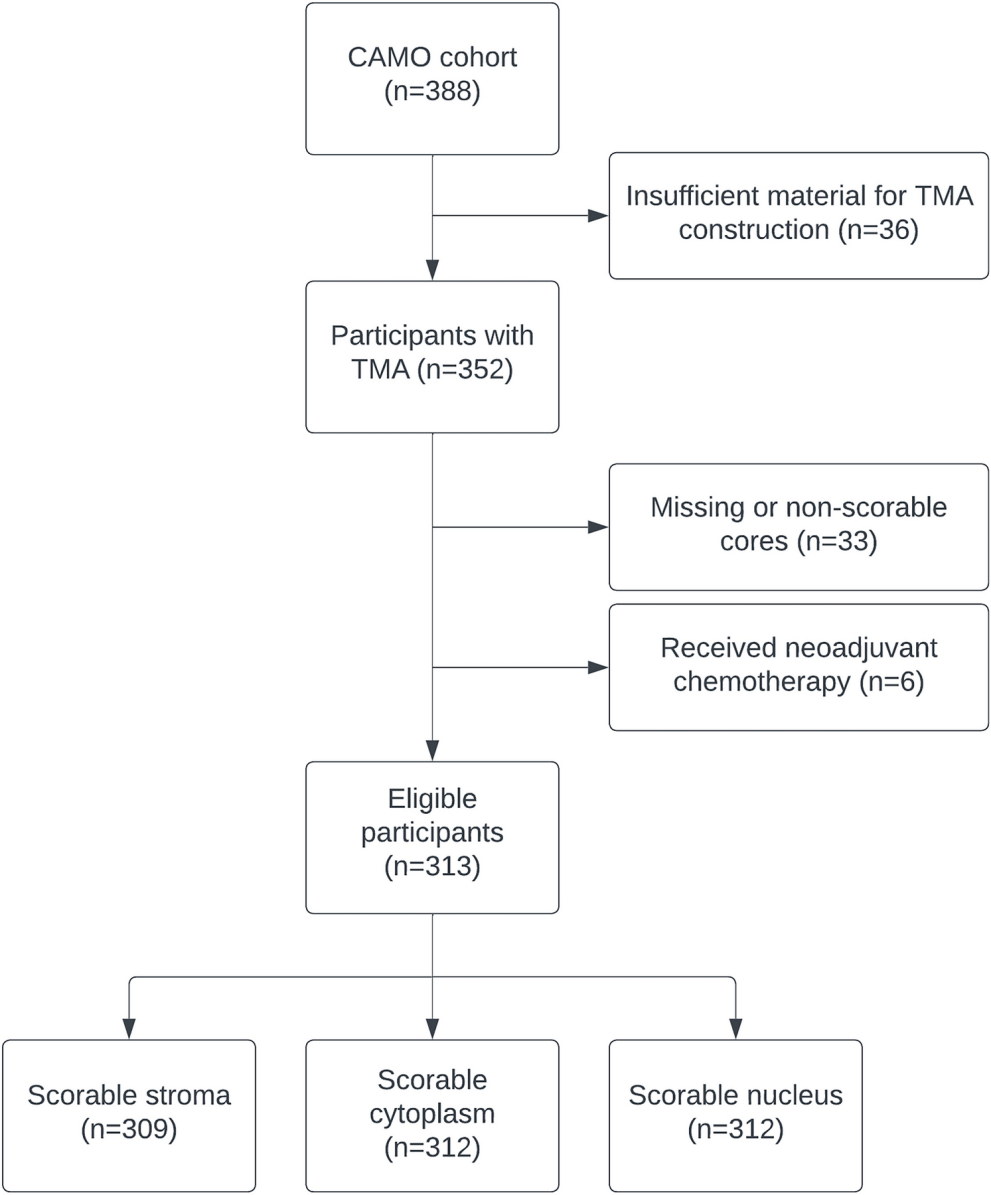
Study population. Inclusion of participants from the CAMO cohort to this study. *TMA* tissue microarray.

From the initial 388 CAMO participants, 69 were excluded for technical reasons, such as missing formalin-fixed paraffin-embedded (FFPE) tissue blocks, too small tumors, fragmentation during TMA construction due to high adipose tissue fraction, or non-scorable tumor cores. Lastly, those who had received neoadjuvant treatment were excluded (n = 6), resulting in a final cohort consisting of 313 participants with scorable tissue cores. Among these participants, 309 had tissue cores with scorable stroma, 312 had scorable cytoplasm and 312 had scorable nuclei (Fig. 1). A total of 259 (82.7%) participants answered the questionnaires as part of the NOWAC cohort before their breast cancer diagnosis, while 54 (17.3%) patients gave this information after diagnosis.

### Definitions and recoding of variables

Tumors were graded based on gland formation, nuclear pleomorphism, and mitotic count as part of routine diagnostic assessment. Expression of estrogen receptor (ER), progesterone receptor (PR) and human epidermal growth factor receptor 2 (HER2) was evaluated in needle biopsies as previously described^25^. The Ki67 expression was analyzed in tumor tissue slides from the surgical specimens and reported as the percentage of positive cancer cell nuclei in the most proliferative parts of the tumors. Molecular subtyping of tumors was done based on the surrogate markers ER, PR, HER2 and Ki67, according to recommendations by the St. Gallen International Expert Consensus^25,27,28^. Information on tumor diameter, lymph node metastasis, distant metastasis and relapse was manually curated from medical records.

Age at menarche, parity, body mass index (BMI), smoking, alcohol consumption, menopausal status, and family history of breast cancer in mother or sister were self-reported in NOWAC questionnaires. Parity was categorized as nulliparous and parous, BMI was grouped into three categories: < 25, 25–30 and > 30, and smoking status of the participants was set to ever or never. In case of missing data on age at menopause, women were classified as pre- or postmenopausal using an age cut-off of 53 years.

### MicroRNA expression in tumor tissue

#### TMA construction

The methodology has previously been reported in detail^29^. Briefly, representative tumor areas were selected on histological slides from the primary tumors by two pathologists (LM and LTRB). A map with coordinates for each patient was made before collecting tissue cores. Several replicate 0.6 mm tissue cores were transferred from each paraffin donor block to a recipient block using a tissue arraying instrument (Beecher Instruments, Silver Spring, MD) and 4 μm sections were prepared (Microm microtome HM355S, Microm, Walldorf, Germany).

#### In situ hybridization

Labeling of miR-20a-5p by ISH was performed in the Ventana Discovery Ultra instrument (Ventana Medical Inc, Marana, AZ, USA). Double‐digoxigenin (DIG) labeled miRCURY LNA detection- and control probes from Exiqon AS, Denmark were used.

Adequate sensitivity level of the ISH method and minimal RNA degradation were confirmed by a control probe targeting U6, a small nuclear RNA component of the spliceosome. A scramble miRNA negative control probe indicated no unspecific staining from reagents or tissues. MiR-20a-5p expression in other tissues than breast cancer was also confirmed by a multi tissue TMA control. Reagents and probes used are shown in Supplementary Table S1.

Briefly, TMA slides were baked at 60 °C overnight and then transferred to the Discovery Ultra for ISH staining. After deparaffinization, heat retrieval and denaturation, probes were hybridized to tissue RNA targets. Stringency washing and blocking of unspecific bindings were performed. The RNA-bound probes were then detected immulogically by binding to alkaline phospatase-conjugated anti-DIG and visualized by substrate enzymatic reactions. Finally, the slides were counterstained, dehydrated through an increasing gradient of ethanol solutions to xylene, and mounted. Details of the optimized ISH protocol are shown in Supplementary Table S2.

#### Semiquantitative scoring

**Fig. 2:**
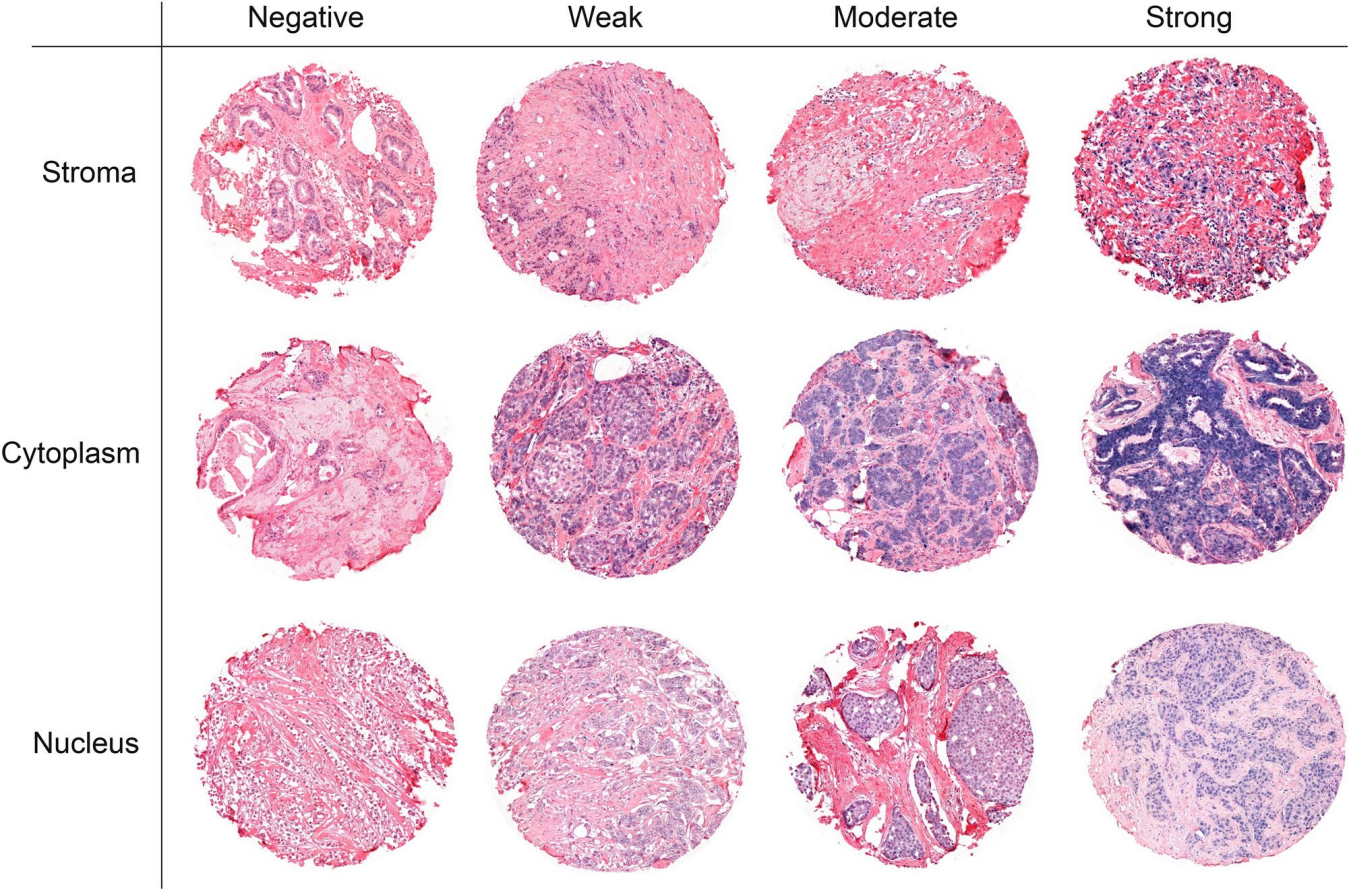
Semiquantitative scoring of miR-20a-5p in tumor tissue. A panel of representative tissue cores with scoring of miR-20a-5p stained by in situ hybridization (ISH) in tumor stromal fibroblasts (stroma), cancer cell cytoplasm (cytoplasm) and cancer cell nuclei (nucleus). Cores were given scores of 0–3 based on the intensity of the staining in each tissue- and subcellular compartment.

The TMAs were digitized using a Panoramic 250 Flash III slide scanner (3DHistech, Budapest, Hungary), and uploaded to the bioimage analysis software QuPath version 0.1.2. In scorable cores, the staining density was scored in a four-tiered ordinal scale (0 = negative, 1 = weak, 2 = moderate and 3 = strong). Cores with representative scoring values are shown in Fig. 2. Each tissue core received one score for each of the three different compartments: tumor stromal fibroblasts, cancer cell cytoplasm and cancer cell nuclei. All samples were anonymized and independently scored by one pathologist (LM) and two researchers (EST and ABD), who were blinded to the scores of the other researchers and the patients’ outcomes. In cases where there was a score discrepancy greater than 1, the slides were re-examined until a consensus was reached. A mean score for each compartment was calculated from all cores of the patient and all examiners. A predetermined scoring value of 2 was used as a cutoff to dichotomize the mean scoring value as high or low. To assess scoring in cancer cell cytoplasm and nucleus combined, the dichotomized scoring categories were combined as either high/high, low/low or mixed.

### Functional in vitro studies

#### Cell lines and culture

The functional properties of miR-20a-5p were evaluated in three different breast cancer cell lines: SK-BR-3 (ATCC^®^ HTB-30), MDA-MB-231 (ATCC^®^ HTB-26) and MCF-7 (ATCC^®^ HTB-22), all derived from metastatic sites (pleural effusions). SK-BR-3 is a HER2-positive cell line, characterized by HER2 overexpression and the absence of ER and PR. MDA-MB-231 is a triple-negative cell line, lacking ER, PR, and HER2 expression. MCF-7 represents the luminal A subtype, exhibiting ER and PR positivity, with low or undetectable levels of HER2^30^.

To reduce the risk of significant changes to the cells due to mutations during the passages, we plated SK-BR-3 cells below passage 20, MDA-MB-231 cells below passage 15 and MCF-7 cells below passage 10. The cells (2 × 10^5^ cells/ml) were cultured in Opti-MEM I (1×) medium without phenol red (catalog# 11058-021, GIBCO, RF, UK), supplemented with 5% of fetal bovine serum (FBS) (catalog# S0415, Biochrom, Berlin, Germany) and Penicillin Streptomycin 1% (catalog# 15140-148, Gibco, NY, USA), in a humidified atmosphere with 5% CO_2_: 95% air, at 37 °C, for 72 h. The culture medium was then replaced by serum-free medium 24 h before the experiments. At the start of the experiments, the cells were 85–90% confluent.

#### Cell transfection

Cells were transiently transfected with hsa-miR-20a-5p Pre-miR™ miRNA Precursor (catalog# AM17100, Thermo Fisher Scientific, USA), alongside the Cy3™ Dye-Labeled Pre-miR Negative Control #1 (catalog# AM17120, Thermo Fisher Scientific, USA) using the transfection reagent Lipofectamine™ RNAiMAX (catalog# 13778075, Thermo Fisher Scientific, USA). Transfected Cy3™ Dye-Labeled Pre-miR Negative Control emits fluorescent light upon UV-light exposure. The transfection efficiency, assessed by fluorescence microscopy, ranged from 80 to 95%.

#### MTT assay for proliferation

Cells were cultured at 5 × 10^3^ cells/well in 96 well plates and then transfected with either hsa-miR-20a-5p Pre-miR™ miRNA Precursor or Cy3™ Dye-Labeled Pre-miR Negative Control. At 0, 1, 2 and 3 days after transfection, cells were treated with 12 mM of [3-(4,5-dimethylthiazol-2-yl)-2,5-diphenyltetrazolium bromide] (MTT, 5 mg/ml) (catalog# M6494, Invitrogen, OR, USA) and incubated for 4 h at 37 °C. The resulting formazan crystals were solubilized by incubating the cells in 0.01 M HCl/SDS (catalog# 28312, Thermo Scientific, IL, USA) at 37 °C overnight and then quantified spectrophotometrically by measuring the absorbance at 570 nm in the CLARIOstar^®^ plate reader (BMG Labtech, Ortenberg, Germany). Three different experiments with four parallel wells were performed for each cell line (Supplementary Information, Fig. S1).

#### Wound healing assay for migration

Cells were cultured at 2 × 10^5^ cells/well in 24 well plates, washed with phosphate buffered saline (PBS) and incubated in a serum-free culture medium containing mitomycin C (10 µg/L), which blocks DNA replication to avoid cell proliferation. The cell monolayer was scraped with a 200 µl sterile pipette tips to create a “wound”, and then washed to remove detached cells and debris. After 4 h, the cells were transfected with either hsa-miR-20a-5p Pre-miR™ miRNA Precursor or Cy3™ Dye-Labeled Pre-miR Negative Control and incubated for 24 h at 37 °C. To measure wound closure, photographs of the same areas of the wound were taken at 0 and 24 h. An inverted optical microscope (Nikon Eclipse TS100) was used to capture images, which were further analyzed by Micrometrics SE Premium 4 software. To determine the extent of cell migration during the 24-h incubation period, the areas occupied by migrated cells were quantified by subtracting the background levels at 0 h.

#### Transwell assay for invasion

Cells (2 × 10^5^) in serum-free culture medium were seeded in ThincertR chambers (Greiner Bio-one, Kremsmünster, Austria) with polyethylene terephthalate membranes (8 mm pore size) pre-coated with 50 ml of phenol red-free Matrigel (Gibco). The chambers were placed in 24-well plates containing culture medium with 5% FBS in the lower chamber. Cells in the upper chambers were transfected with either hsa-miR-20a-5p Pre-miR™ miRNA Precursor or Cy3™ Dye-Labeled Pre-miR Negative Control and incubated for 48 h at 37 °C. The chambers were washed with 10 mM PBS, fixed in 4% paraformaldehyde for 30 min, and stained with 0.2% crystal violet for 10 min. Non-invading cells from the upper membrane surface were removed with a cotton swab. Invaded cells on the lower membrane surface were photographed using an inverted optical microscope (Nikon Eclipse TS1000). Images of three random microscope fields were captured in duplicate. To quantify invasion, the number of cells on the membranes were counted using Image J software (National Institutes of Health, Bethesda, MD, USA).

### Statistical methods

We calculated interobserver reliability between miR-20a-5p scores by applying two-way random effects models with absolute agreement definition. The correlations between miR-20 expression levels in different compartments were calculated using Spearman’s correlation coefficients.

For normally distributed data, independent samples t-test was used to compare means between two independent groups, and one-way ANOVA for three or more groups. For variables where the assumption of normality was not met, Mann–Whitney U test was used to compare medians between two independent groups, and the Kruskal–Wallis test for three or more groups. The chi-squared test was used to check for associations between categorical variables. Logistic regression was used to model associations of miR-20 expression levels in different compartments with breast cancer risk factors and clinicopathological parameters. The results from the logistic regression analyses were presented as odds ratios (OR) with 95% confidence intervals (CI). In the absence of prior knowledge regarding potentially confounding factors, no adjustments were made in the statistical analysis.

We examined the relationship between miR-20a-5p expression levels and anthropometric, lifestyle and reproductive factors, using smoking, alcohol, parity, BMI, and menopausal status at diagnosis as predictor variables, with miR-20a-5p expression as the outcome. In analyzing the relationship between miR-20a-5p expression levels and clinicopathological factors, we used miR-20a-5p expression as the predictor variable for all analyses except tumor grade and molecular subgroup, which were used as the predictor variables with miR-20a-5p expression as the outcome.

Restricted cubic splines with four knots were used to assess possible non-linear relationship between stromal, cytoplasmic and nuclear miR-20a-5p expression and the outcome variables relapse, lymph node metastasis and distant metastasis. Locations of knots were based on Harrell’s recommended percentiles of the mean miR-20a-5p scoring values^31^. The restricted cubic splines for all three compartments were modeled with four knots positioned at the 5th, 35th, 65th and 95th percentiles of the mean scoring values. A Wald-type test was used to assess if the coefficients of the second and third splines were equal to zero.

For the functional in vitro studies, differences between transfected and control experiments were analyzed using independent samples t-tests.

Statistical analyses were done in STATA/MP version 17.0 (Stata Corp, College Station, TX, USA), GraphPad Prism 9 (GraphPad Software, Boston, MA, USA) and Microsoft Excel (Microsoft Office 365, Microsoft Corp., Redmond, WA, USA). A p-value of 0.05 was considered statistically significant for all analyses.

### Language revision

ChatGPT version 4o (OpenAI Inc, San Fransisco, CA, USA) was used to improve grammar, sentence structure, and readability of minor parts of the manuscript. All AI-generated suggestions were critically evaluated to ensure that the original meaning of the text was preserved. Final language revision was done by a native English speaker.

## Results

### Patient characteristics

Patient characteristics for CAMO cohort participants have previously been described in detail^25^. Characteristics for the subset of patients used in this study are presented in Table 1. In short, the median age at diagnosis was 56 years, most cancers were classified as tumor grade 2 (42.3%) and were of the luminal A subtype (62.2%). A total of 40 (12.8%) tumors were of the basal-like subtype. Median tumor diameter was 16 mm, and 32.0% of women underwent mastectomy. A total of 258 (82.4%) tumors were hormone receptor (HR) positive, and 38 (12.2%) were HER2 positive. At diagnosis, 30.2% were diagnosed with lymph node metastasis. At any point during follow-up, 25 (8%) experienced locoregional relapse and 34 (11.0%) experienced distant metastasis. The mean follow-up time was 165.6 months.

**Table 1:** Patient characteristics.

	Distribution	Missing, n (%)
Age at diagnosis, median (range)	56 (35–68)	0
Tumor grade, n (%)	312	1 (0.3)
1	98 (31.4)	
2	132 (42.3)	
3	82 (26.3)	
Molecular subgroup, n (%)	312	1 (0.3)
Luminal A	194 (62.2)	
Luminal B	63 (20.2)	
HER2 +	15 (4.8)	
Basal-like	40 (12.8)	
Tumor diameter in millimeters, median (range)	16 (1–92)	0
Surgery, n (%)	313	0
Lumpectomy	213 (68.1)	
Mastectomy	100 (32.0)	
Lymph node metastasis, n (%)	311	2 (0.6)
Yes	94 (30.2)	
No	217 (69.8)	
Distant metastasis^a^, n (%)	310	3 (1.0)
Yes	34 (11.0)	
No	276 (89.0)	
Relapse, n (%)	311	2 (0.6)
Yes	25 (8.0)	
No	286 (92.0)	
ER positive, n (%)	313	0
Yes	253 (80.8)	
No	60 (19.2)	
PR positive, n (%)	303	10 (3.2)
Yes	203 (67.0)	
No	100 (33.0)	
HER2 positive, n (%)	312	1 (0.3)
Yes	38 (12.2)	
No	274 (87.8)	
Menopausal status at diagnosis	313	0
Post-menopausal	219 (70.0)	
Pre-menopausal	94 (30.0)	
Age at menarche, mean (SD)	13.0 (1.3)	4 (1.3)
Parity	313	0
0	32 (10.2)	
≥ 1	281 (89.8)	
Body mass index, n (%)	306	7 (2.2)
< 25	182 (59.5)	
25–30	95 (31.1)	
> 30	29 (9.5)	
Smoking status at first questionnaire, n (%)	312	2 (0.6)
Never	108 (34.6)	
Ever	204 (65.4)	

*ER* estrogen receptor, *HER2* human epidermal growth factor receptor 2, *PR* progesterone receptor, *IQR* interquartile range, *SD* standard deviation.

^a^Distant metastasis at any time point.

### MiRNA expression in tumor tissue

The scoring agreement between the three researchers was as follows: stroma 67%; cytoplasm 80% and nucleus 78%. We observed a high positive correlation between scoring values in nucleus and stroma (r_s_ = 0,71), cytoplasm and stroma (r_s_ = 0,67), and cytoplasm and nucleus (r_s_ = 0,67). The proportion of tumors classified as having high expression of miR-20 was as follows: stroma 14.9%; cytoplasm 23.4%; and nucleus 57.7% (Table 2). The expression of mir-20a-5p was high in all compartments in 9% of the tumors.

**Table 2:** MiR-20a-5p expression levels in different compartments.

	Stroma n = 309		Cytoplasm n = 312		Nucleus n = 312	
	High	Low	High	Low	High	Low
Distribution, n (%)	46 (14.9)	263 (85.1)	73 (23.4)	239 (76.6)	180 (57.7)	132 (42.3)
Missing, n (%)	4 (1.3)		1 (0.3)		1 (0.3)	

MiR-20a-5p expression in tumor stromal fibroblasts (stroma), cancer cell cytoplasm (cytoplasm), and cancer cell nucleus (nucleus).

### Associations of miR-20a-5p expression with breast cancer risk factors and clinicopathological features

MiR-20a-5p expression levels were assessed in relation to demographic, anthropometric, lifestyle, reproductive and clinicopathological factors (Tables 3, 4, 5, 6, 7).

**Table 3:** Associations of miR-20a-5p expression levels with breast cancer risk factors.

	Stroma		Cytoplasm		Nucleus		Cytoplasm and nucleus		
	High	Low	High	Low	High	Low	High/high	Low/low	Mixed
Age at diagnosis, y, median (IQR)	52 (14)	57 (9)	54 (13)	57 (8)	56 (13)	57 (7)	54 (16)	57 (7)	57 (11)
p	0.009*		0.049*		0.49		0.15		
BMI, n (%)									
< 25	31 (68.9)	149 (58.0)	46 (63.0)	135 (58.2)	116 (65.5)	65 (50.8)	45 (63.4)	64 (50.8)	72 (66.7)
25–30	12 (26.7)	82 (31.9)	22 (30.1)	73 (31.5)	46 (26.0)	49 (38.3)	21 (29.6)	48 (38.1)	26 (24.1)
> 30	2 (4.4)	26 (10.1)	5 (6.0)	24 (10.3)	15 (8.5)	14 (10.9)	5 (7.0)	14 (11.8)	10 (9.3)
p	0.30		0.62		0.033*		0.12		
Age at menarche, y, mean (SD)	12.9 (1.1)	13.5 (1.4)	13.1 (1.3)	13.0 (1.3)	13.0 (1.3)	13.1 (1.3)	13.0 (1.3)	13.1 (1.3)	12.9 (1.3)
p	0.26		0.60		0.19		0.99		
Menopausal status**, n (%)									
Post	23 (50.0)	194 (73.8)	43 (58.9)	176 (73.6)	119 (66.1)	100 (75.8)	41 (57.8)	98 (75.4)	80 (72.1)
Pre	23 (50.0)	69 (26.2)	30 (41.1)	63 (26.4)	61 (33.9)	32 (24.2)	30 (42.3)	32 (24.6)	31 (27.9)
p	0.001*		0.016*		0.07		0.028*		
Parity, n (%)									
Parous	42 (91.3)	236 (89.7)	68 (93.2)	212 (88.7)	160 (88.9)	120 (90.9)	66 (93.0)	118 (90.8)	96 (86.5)
Nulliparous	4 (8.7)	27 (10.3)	5 (6.9)	27 (11.3)	20 (11.1)	12 (9.1)	5 (7.0)	12 (9.2)	15 (13.5)
p	0.74		0.27		0.56		0.33		
OC use, n (%)									
Yes	25 (55.6)	159 (62.9)	44 (62.0)	142 (61.7)	111 (63.4)	75 (59.5)	43 (62.3)	74 (59.7)	69 (63.9)
No	20 (44.4)	94 (37.2)	27 (38.0)	88 (38.3)	64 (36.6)	51 (40.5)	26 (37.7)	50 (40.3)	39 (36.1)
p	0.35		0.97		0.49		0.80		
Alcohol consumption, g, median (IQR)	2.1 (2.4)	1.6 (4.7)	2.1 (2.7)	1.6 (4.1)	1.7 (3.5)	1.6 (5.0)	2.1 (2.7)	1.6 (5.1)	1.5 (3.1)
p	0.88		0.18		0.39		0.15		
Smoking, n (%)									
Never	14 (30.4)	92 (35.1)	24 (32.9)	84 (35.3)	65 (36.1)	43 (32.8)	24 (33.8)	43 (33.3)	41 (36.9)
Ever	32 (69.6)	170 (64.9)	49 (67.1)	154 (64.7)	115 (63.9)	88 (67.2)	47 (66.2)	86 (66.7)	70 (63.1)
p	0.54		0.70		0.55		0.83		
Mother with BC, n (%)									
Yes	1 (2.2)	13 (5.3)	5 (7.1)	9 (4.0)	9 (5.3)	5 (4.0)	5 (7.4)	5. (4.1)	4 (3.8)
No	44 (97.8)	234 (94.7)	65 (92.9)	216 (96.0)	162 (94.7)	119 (96.0)	63 (92.7)	117 (95.9)	101 (96.2)
p	0.38		0.28		0.62		0.51		
Sister with BC, n (%)									
Yes	1 (2.4)	4 (1.8)	1 (1.6)	4 (2.0)	4 (2.6)	1 (0.9)	1 (1.7)	1 (0.9)	3 (3.2)
No	40 (97.6)	218 (98.2)	60 (98.4)	201 (98.1)	148 (97.4)	113 (99.1)	58 (98.3)	111 (99.1)	92 (96.8)
p	0.78		0.88		0.30		0.49		

Associations between breast cancer risk factors and miR-20a-5p expression in tumor stromal fibroblasts (stroma), cancer cell cytoplasm (cytoplasm), cancer cell nuclei (nucleus), and cancer cell compartments combined. *BC* breast cancer, *BMI* body mass index, *g* grams, *IQR* interquartile range, *OC* oral contraceptive, *SD* standard deviation, *y* years. *Statistically significant (p < 0.05). **Menopausal status at diagnosis.

**Table 4:** Associations of miR-20a-5p expression levels with clinicopathological features.

	Stroma		Cytoplasm		Nucleus		Cytoplasm and nucleus		
	High	Low	High	Low	High	Low	High/high	Low/low	Mixed
Tumor size, cm, median (IQR)	1.5 (1.3)	1.7 (1.3)	1.5 (1.5)	1.7 (1.2)	1.5 (1.3)	1.9 (1.3)	1.5 (1.5)	1.9 (1.3)	1.5 (1.2)
p	0.20		0.63		0.030*		0.06		
Tumor grade, n (%)									
1	12 (26.1)	85 (32.4)	24 (32.9)	74 (31.1)	60 (33.5)	38 (28.8)	22 (31.0)	36 (27.7)	40 (36.4)
2	19 (41.3)	110 (42.0)	23 (31.5)	109 (45.8)	67 (37.4)	65 (49.2)	23 (32.4)	65 (50.0)	44 (40.0)
3	15 (32.6)	67 (25.6)	26 (35.6)	55 (23.1)	52 (29.1)	29 (22.0)	26 (36.6)	29 (22.3)	26 (23.6)
p	0.54		0.047*		0.11		0.06		
Molecular subgroup, n (%)									
Luminal A	26 (56.5)	165 (63.0)	41 (55.4)	152 (63.9)	113 (63.1)	80 (60.6)	40 (56.3)	79 (60.8)	74 (67.3)
Luminal B	10 (21.7)	53 (20.2)	15 (20.3)	49 (20.6)	36 (20.1)	27 (20.5)	13 (18.3)	26 (20.0)	24 (21.8)
HER2-enriched	2 (4.4)	13 (5.0)	4 (5.4)	11 (4.6)	6 (3.4)	9 (6.8)	4 (5.6)	9 (6.9)	2 (1.8)
Basal-like	8 (17.4)	31 (11.8)	14 (18.9)	26 (10.9)	24 (13.4)	16 (12.1)	14 (19.7)	16 (12.3)	10 (9.1)
p	0.73		0.30		0.56		0.21		
Ki67, n (%)									
High	19 (47.5)	68 (30.9)	27 (42.9)	60 (30.2)	58 (37.2)	29 (27.4)	27 (43.6)	29 (27.6)	31 (32.6)
Low	21 (52.5)	152 (69.1)	36 (57.1)	139 (69.9)	98 (62.8)	77 (72.6)	35 (56.5)	76 (72.4)	64 (67.4)
p	0.041*		0.06		0.10		0.11		
ER + , n (%)									
Yes	36 (78.3)	214 (81.4)	54 (74.0)	198 (82.9)	148 (82.2)	104 (78.8)	53 (74.7)	103 (79.2)	96 (85.5)
No	10 (21.7)	49 (18.7)	19 (26.0)	41 (17.2)	32 (17.8)	28 (21.2)	18 (25.4)	27 (20.8)	15 (13.5)
p	0.62		0.09		0.45		0.12		
PR + , n (%)									
Yes	29 (65.9)	173 (67.8)	49 (68.1)	153 (66.5)	118 (68.2)	84 (65.1)	47 (67.1)	82 (64.6)	73 (69.5)
No	15 (34.1)	82 (32.2)	23 (31.9)	77 (33.5)	55 (31.8)	45 (34.9)	23 (32.9)	45 (35.4)	32 (30.5)
p	0.80		0.81		0.57		0.73		
HER2 + , n (%)									
Yes	6 (13.0)	32 (12.2)	7 (9.6)	31 (13.0)	20 (11.2)	18 (13.6)	6 (8.5)	17 (13.1)	15 (13.6)
No	40 (87.0)	230 (87.8)	66 (90.4)	207 (87.0)	159 (88.8)	114 (86.4)	65 (91.6)	113 (86.9)	95 (86.4)
p	0.88		0.43		0.51		0.54		
Relapse, n (%)									
Yes	8 (17.4)	17 (6.5)	7 (9.7)	18 (7.6)	18 (10.1)	7 (5.3)	7 (10.0)	7 (5.4)	11 (9.9)
No	38 (82.6)	244 (93.5)	65 (90.3)	220 (92.4)	161 (89.9)	124 (94.7)	63 (90.0)	122 (95.6)	100 (90.1)
p	0.013*		0.55		0.13		0.35		
Lymph node metastasis, n (%)									
Yes	16 (34.8)	76 (29.1)	16 (21.9)	78 (32.9)	46 (25.7)	48 (36.6)	16 (22.5)	48 (37.2)	30 (27.3)
No	30 (65.2)	185 (70.9)	57 (78.1)	159 (67.1)	133 (74.3)	83 (63.4)	55 (77.5)	81 (62.8)	80 (72.7)
p	0.44		0.07		0.038*		0.07		
Distant metastasis, n (%)									
Yes	5 (11.1)	28 (10.7)	8 (11.1)	26 (11.0)	16 (9.0)	18 (13.7)	8 (11.4)	18 (14.0)	8 (7.3)
No	40 (88.9)	233 (89.3)	64 (88.9)	211 (89.0)	162 (91.0)	113 (86.3)	62 (88.6)	111 (86.1)	102 (92.7)
p	0.94		0.97		0.19		0.26		

Associations between clinicopathological features and miR-20a-5p expression in tumor stromal fibroblasts (stroma), cancer cell cytoplasm (cytoplasm), cancer cell nuclei (nucleus), and cancer cell compartments combined. *ER* estrogen receptor, *HER2* human epidermal growth factor receptor 2, *IQR* interquartile range, *PR* progesterone receptor, *SD* standard deviation. *Statistically significant (p < 0.05).

**Table 5:** Logistic regression analysis of the association between mir-20a-5p expression levels in stroma, in relation to clinicopathological parameters and breast cancer risk factors.

	N	Odds ratio	95% Confidence interval	p-value
Tumor size	309	0.90	0.67–1.20	0.48
Tumor grade				
1	97	Ref	Ref	Ref
2	129	1.22	0.56–2.66	0.61
3	82	1.59	0.70–3.61	0.27
Molecular subgroup				
Luminal A	191	Ref	Ref	Ref
Luminal B	63	1.20	0.54–2.64	0.66
HER2-enriched	15	0.98	0.21–4.58	0.98
Basal like	39	1.64	0.68–3.95	0.27
Estrogen receptor				
Negative	59	Ref	Ref	Ref
Positive	250	0.82	0.38–1.77	0.62
Progesterone receptor				
Negative	97	Ref	Ref	Ref
Positive	202	0.92	0.47–1.80	0.80
HER2				
Negative	270	Ref	Ref	Ref
Positive	38	1.08	0.42–2.74	0.88
Ki67				
Low	173	Ref	Ref	Ref
High	87	2.02	1.02–4.01	0.043
Lymph node metastasis				
No	215	Ref	Ref	Ref
Yes	92	1.30	0.67–2.52	0.44
Distant metastasis^a^				
No	273	Ref	Ref	Ref
Yes	33	1.04	0.38–2.85	0.94
Relapse				
No	282	Ref	Ref	Ref
Yes	25	3.02	1.22–7.49	0.017
Menopausal status^b^				
Post	217	Ref	Ref	Ref
Pre	92	2.81	1.48–5.33	0.002
BMI				
Low	274	Ref	Ref	Ref
High	28	0.41	0.09–1.81	0.24
Parity				
Nulliparous	31	Ref	Ref	Ref
Parous	278	1.20	0.40–3.61	0.74
Oral contraceptives				
Never	114	Ref	Ref	Ref
Ever	184	0.74	0.39–1.40	0.36
Alcohol consumption	302	0.99	0.92–1.07	0.76
Smoking				
Never	106	Ref	Ref	Ref
Ever	202	1.24	0.63–2.44	0.54

*HER2* Human epidermal growth factor receptor 2. ^a^At any time, ^b^At diagnosis.

**Table 6:** Logistic regression analysis of mir-20a-5p expression levels in cytoplasm, in relation to clinicopathological parameters and breast cancer risk factors.

	N	Odds ratio	95% Confidence interval	p-value
Tumor size	312	0.93	0.74–1.17	0.54
Tumor grade				
1	98	Ref	Ref	Ref
2	132	0.65	0.34–1.24	0.19
3	81	1.46	0.76–2.81	0.26
Molecular subgroup				
Luminal A	193	Ref	Ref	Ref
Luminal B	63	1.06	0.53–2.11	0.87
HER2-enriched	15	1.35	0.41–4.45	0.62
Basal like	40	2.00	0.96–4.17	0.07
Estrogen receptor				
Negative	60	Ref	Ref	Ref
Positive	252	0.59	0.32–1.10	0.10
Progesterone receptor				
Negative	100	Ref	Ref	Ref
Positive	202	1.07	0.61–1.89	0.81
HER2				
Negative	273	Ref	Ref	Ref
Positive	38	0.71	0.30–1.68	0.44
Ki67				
Low	175	Ref	Ref	Ref
High	87	1.74	0.97–3.11	0.06
Lymph node metastasis				
No	216	Ref	Ref	Ref
Yes	94	0.57	0.31–1.06	0.08
Distant metastasis^a^				
No	275	Ref	Ref	Ref
Yes	34	1.01	0.44–2.35	0.97
Relapse				
No	285	Ref	Ref	Ref
Yes	25	1.32	0.53–3.30	0.56
Menopausal status^b^				
Post	219	Ref	Ref	Ref
Pre	93	1.95	1.13–3.37	0.017
BMI				
Low	276	Ref	Ref	Ref
High	29	0.64	0.23–1.74	0.38
Parity				
Nulliparous	32	Ref	Ref	Ref
Parous	280	1.73	0.64–4.67	0.28
Oral contraceptives				
Never	115	Ref	Ref	Ref
Ever	186	1.01	0.58–1.75	0.97
Alcohol	306	1.03	0.98–1.09	0.25
Smoking				
Never	108	Ref	Ref	Ref
Ever	203	1.11	0.64–1.94	0.70

*HER2* Human epidermal growth factor receptor 2. ^a^At any time, ^b^At diagnosis.

**Table 7:** Logistic regression analysis of miR-20a-5p expression levels in nucleus, in relation to clinicopathological parameters and breast cancer risk factors.

	N	Odds ratio	95% Confidence interval	p-value
Tumor size	312	0.81	0.67–0.99	0.039
Tumor grade				
1	98	Ref	Ref	Ref
2	132	0.65	0.38–1.11	0.12
3	81	1.14	0.62–2.09	0.68
Molecular subgroup				
Luminal A	193	Ref	Ref	Ref
Luminal B	63	0.94	0.53–1.68	0.84
HER2-enriched	15	0.47	0.16–1.38	0.17
Basal like	40	1.06	0.53–2.13	0.87
Estrogen receptor				
Negative	60	Ref	Ref	Ref
Positive	252	1.25	0.71–2.19	0.45
Progesterone receptor				
Negative	100	Ref	Ref	Ref
Positive	202	1.15	0.71–1.86	0.57
HER2				
Negative	273	Ref	Ref	Ref
Positive	38	0.80	0.40–1.57	0.51
Ki67				
Low	175	Ref	Ref	Ref
High	87	1.57	0.92–2.69	0.10
Lymph node metastasis				
No	216	Ref	Ref	Ref
Yes	94	0.60	0.37–0.97	0.039
Distant metastasis^a^				
No	275	Ref	Ref	Ref
Yes	34	0.62	0.30–1.27	0.19
Relapse				
No	285	Ref	Ref	Ref
Yes	25	1.98	0.80–4.89	0.14
Menopausal status^b^				
Post	219	Ref	Ref	Ref
Pre	93	1.60	0.97–2.65	0.07
BMI				
Low	276	Ref	Ref	Ref
High	29	0.75	0.35–1.62	0.47
Parity				
Nulliparous	32	Ref	Ref	Ref
Parous	280	0.80	0.38–1.70	0.56
Oral contraceptives				
Never	115	Ref	Ref	Ref
Ever	186	1.18	0.74–1.89	0.49
Alcohol	306	0.99	0.94–1.04	0.72
Smoking				
Never	108	Ref	Ref	Ref
Ever	203	0.86	0.54–1.39	0.55

*HER2* Human epidermal growth factor receptor 2. ^a^At any time, ^b^At diagnosis.

For tumor stromal fibroblasts (Tab. 5), we observed a significantly lower median age at diagnosis in women with high expression of miR-20a-5p compared to the group with low expression (p = 0.009). Stromal miR-20a-5p expression was significantly associated with menopausal status (p = 0.001), Ki67 expression (p = 0.041), and relapse (p = 0.013). Logistic regression revealed an association between stromal miR-20a-5p expression and menopausal status (OR 2.81, 95% CI 1.48–5.33), indicating that women who were premenopausal at diagnosis had almost three times higher odds of having high stromal miR-20a-5p expression compared to postmenopausal women. A high Ki67 expression was associated with two-fold higher odds of having a high stromal miR-20a-5p expression, compared to low Ki67 expression (OR 2.02, 95% CI 1.02–4.01). Notably, we found three-fold higher odds of relapse associated with high compared to low stromal miR-20a-5p expression (OR 3.02, 95% CI 1.22–7.49).

For cancer cell cytoplasm (Tab. 6), we observed a significantly lower median age at diagnosis (p = 0.049) in women with high expression of miR-20a-5p compared to the group with low expression. Cytoplasmic miRNA expression was significantly associated with menopausal status (p = 0.016) and tumor grade (p = 0.047). Women who were premenopausal at diagnosis had higher odds of having a high cytoplasmic miR-20a-5p expression, compared to postmenopausal women (OR 1.95, 95% CI 1.13–3.37). Although not significant at the 5% level, our results suggest that a high cytoplasmic miR-20a-5p expression may be associated with a two-fold increase in odds of having a basal like subtype (OR 2.00, 95% CI 0.96–4.17). Similarly, suggestive associations were observed between high cytoplasmic miR-20a-5p expression and high Ki67 expression (OR 1.74, 95% CI 0.97–3.11) and, conflictingly, lower odds of lymph node metastasis (OR 0.57, 95% CI 0.31–1.06).

For cancer cell nucleus (Tab. 7), we observed an association with BMI (p = 0.033) and lymph node metastasis (p = 0.038). Logistic regression revealed an inverse association between tumor size and nuclear miR-20a-5p expression (OR 0.81, 95% CI 0.67–0.99), indicating that women with smaller tumors had increased odds of a high nuclear miR-20a-5p expression. There was also an inverse relationship between nuclear miR-20a-5p expression and odds of lymph node metastasis (OR 0.60, 95% CI 0.37–0.97), indicating that women with a high nuclear miR-20a-5p expression had 40% lower odds of lymph node metastasis.

The combined expression of miR-20a-5p in cancer cell nucleus and cytoplasm was significantly associated with menopausal status (p = 0.028).

**Fig. 3:**
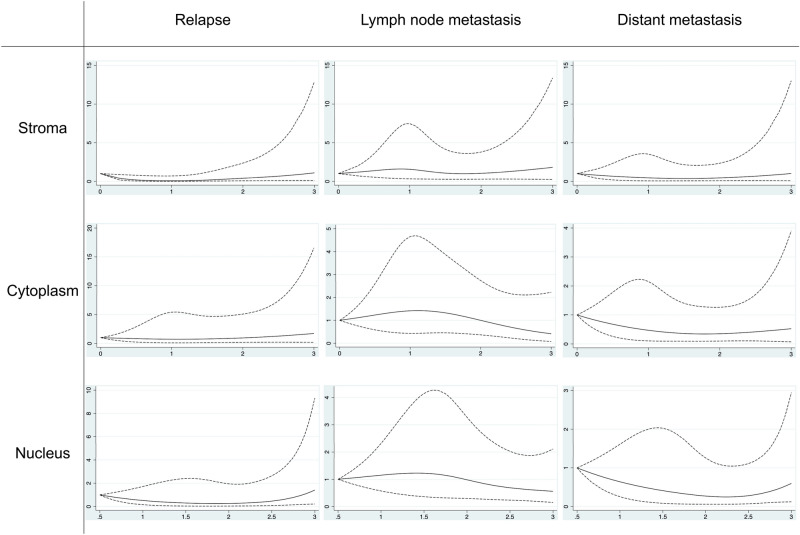
Restricted cubic splines for miR-20a-5p expression levels in different compartments, in relation to clinical outcomes. Spline regression models for stromal, cytoplasmic, and nuclear miR-20a-5p expression levels (scoring values, x-axis) in relation to odds of relapse, lymph node metastasis, and distant metastasis (y-axis). Solid lines: odds ratio, dashed lines: 95% confidence interval.

The associations between the miR-20a-5p scoring values from each tissue- and subcellular compartment and the study outcomes relapse, lymph node metastasis and distant metastasis, estimated from restricted cubic splines models are presented in Fig. 3. We identified a non-linear association between stromal miR-20a-5p expression level and odds of relapse (p = 0.029, Fig. 3). For scoring values up to 2, there was a lower OR for relapse compared to a scoring value of 0. For scores higher than 2, there was an increasing OR for relapse with higher stromal miR-20a-5p expression.

### Functional in vitro studies

**Fig. 4:**
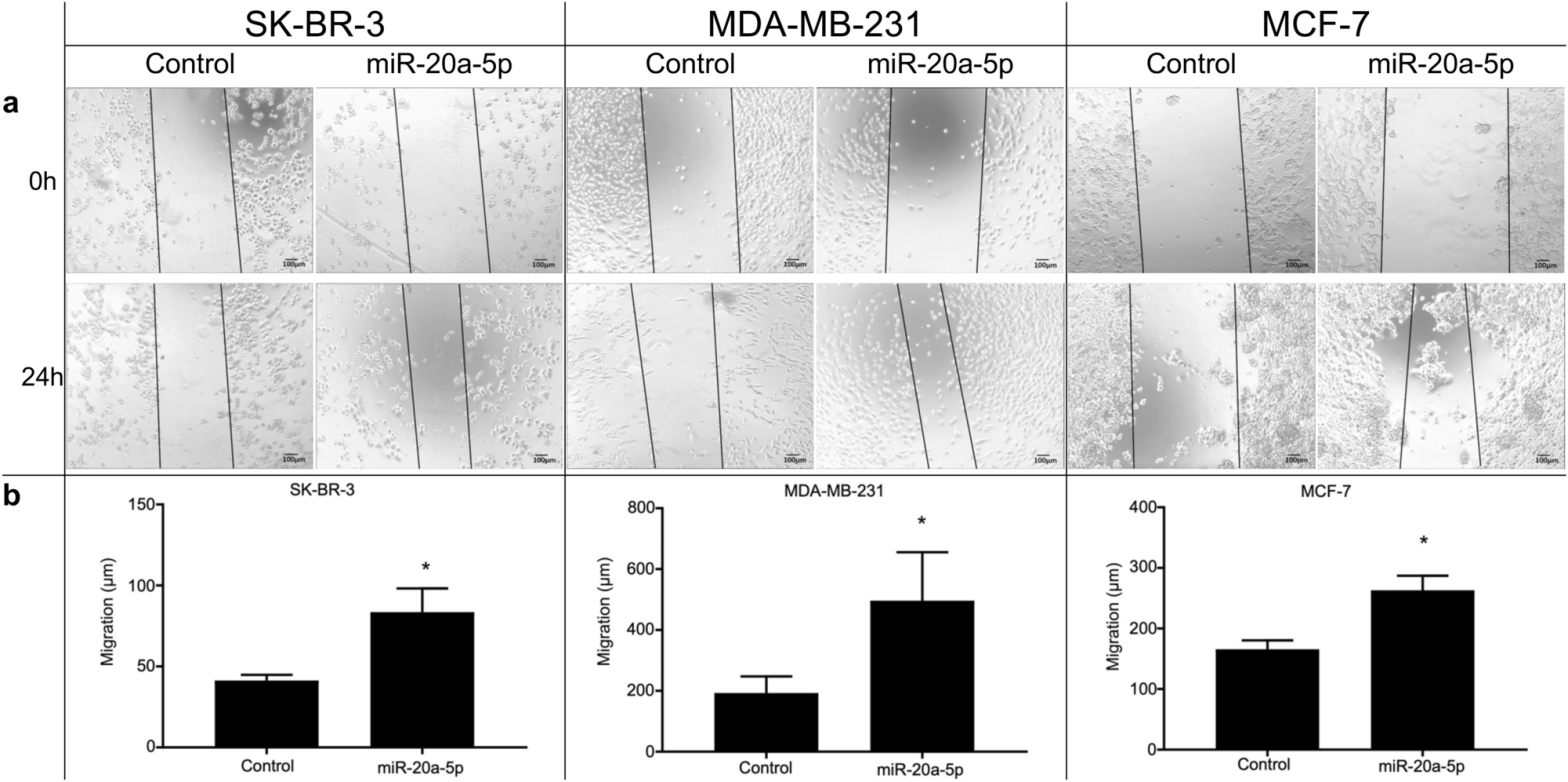
Migration assays in three cell lines. (**a**) Wound healing assay to assess the effects of miR-20a-5p transfection on migration in the breast cancer cell lines SK-BR-3, MDA-MB-231, and MCF-7. Size bars indicate 100 µm. (**b**) The box plots represent the mean distance of migrated cells (µm ± SEM) in three independent experiments. *Significantly different from control.

**Fig. 5:**
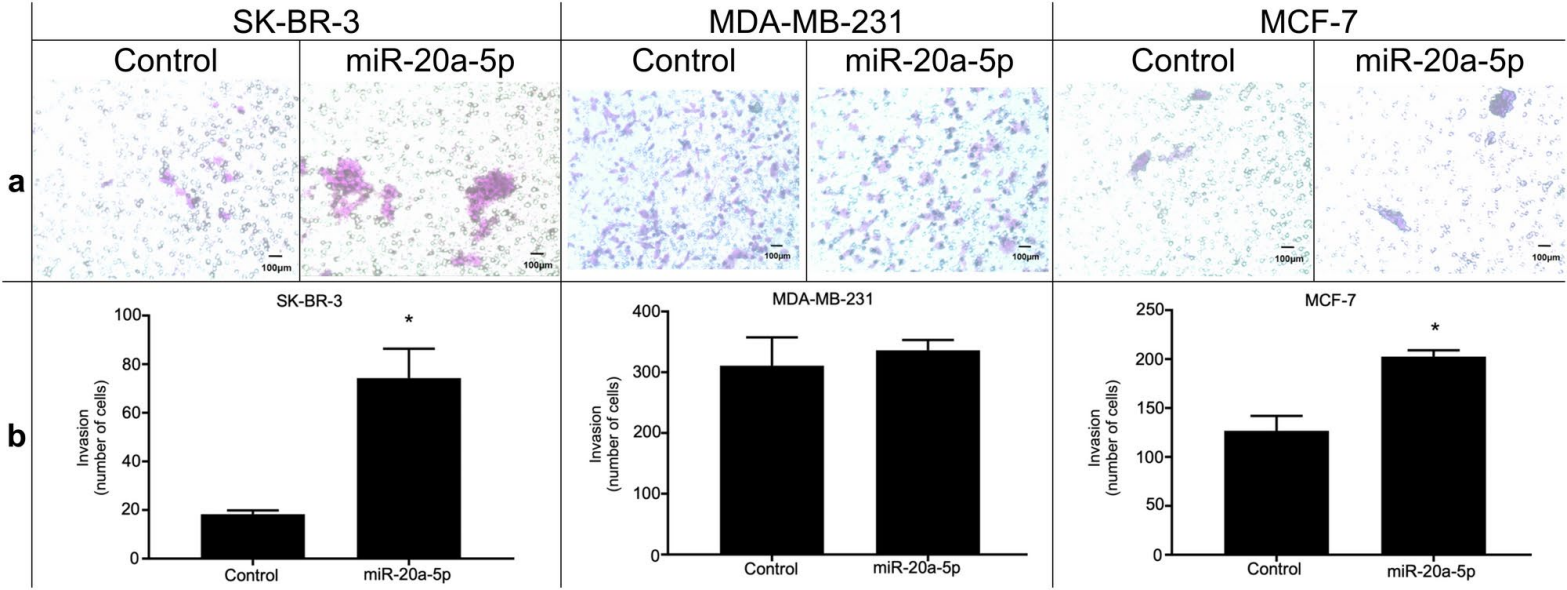
Invasion assays in three cell lines. (**a**) Transwell assay to assess the effects of miR-20a-5p transfection on invasiveness in the breast cancer cell lines SK-BR-3, MDA-MB-231, and MCF-7. Size bars indicate 100 µm. (**b**) The box plots represent the mean number of cells (± SEM) in three independent experiments. *Significantly different from control.

None of the cell lines showed an increase in proliferation upon transfection with miR-20a-5p (Supplementary Information, Fig. S1)**,** according to the proliferation assay. In contrast, wound healing assays demonstrated increased cell migration after 24 h in all cell lines overexpressing miR-20a-5p, compared to controls (Fig. 4). Finally, invasion assays demonstrated that miR-20a-5p overexpression increased invasiveness in both SK-BR-3 and MCF-7 cells, compared to negative controls (Fig. 5).

## Discussion

In this study, we investigated the expression levels and in vitro functional role of miR-20a-5p in breast cancer. The expression level was evaluated in three separate tissue- and subcellular compartments in breast cancer surgical specimens, and we explored its associations with breast cancer risk factors and clinicopathological features. Further, three breast cancer cell lines were used to explore the effect of miR-20a-5p on proliferation, migration and invasion.

Our main findings point to a potential role of miR-20a-5p in more aggressive tumors, however, associations vary according to cell type and subcellular compartment. At large, the in vitro experiments support these findings.

This study demonstrates an association between stromal miR-20a-5p expression and a more aggressive cancer. Women with high stromal miR-20a-5p expression had significantly increased odds of relapse in logistic regression analysis. This association was confirmed using restricted cubic splines, which additionally revealed a non-linear trend. Specifically, ORs for relapse were low for scoring values up to 2, after which they increased progressively from scores of 2 and above (Fig. 3). Despite the wide CI observed in this analysis, the findings remain intriguing and warrant further investigation. The wide CI may be attributed to several factors, including the relatively small sample size, few cases of relapse, and biological variability. Moreover, compared to low expression, tumors with a high stromal miR-20a-5p expression had significantly higher odds of a high Ki67 expression. These findings are particularly intriguing, given the well-established significance of the tumor microenvironment in breast cancer progression^32^.

In lung cancer, it has been demonstrated that miR-20a is transferred from cancer-associated fibroblasts (CAFs) to tumor cells through exosomes^33^. Exosomes are small membrane-enclosed particles that can be secreted by various cell types and internalized by cancer cells, thereby facilitating intercellular communication within the tumor microenvironment^34^. CAF-derived, exosomal miR-20a was shown to upregulate PD-L1 and inhibit PTEN, thereby promoting proliferation and chemoresistance in lung cancer cells^33^. Similar mechanisms have been observed in breast cancer. CAF-derived exosomal miRNAs have been directly linked to ER-repression in breast cancer cells^35^, and to hormonal therapy resistance in luminal breast cancer models^36^. Furthermore, miR-20a-5p has been identified as highly expressed in exosomes from MDA-MB-23 breast cancer cells, and to promote osteoclast proliferation and differentiation through exosome-mediated transfer and binding to target genes, suggesting its role in bone metastasis via tumor cell and bone microenvironment crosstalk^15^. Hence, in vitro studies suggest that crosstalk between fibroblasts and tumor cells, facilitated by exosome-mediated miRNA-transfer, may provide a possible explanation for the observed association of high stromal expression of miRNA-20a-5p and a potentially more aggressive tumor phenotype.

Interestingly, our results demonstrate that cytoplasmic expression of miR-20a-5p in cancer cells is associated with tumor grade, and that high cytoplasmic miR-20a-5p may be associated with high Ki67 and an increased odds of having a basal-like subtype, which is generally recognized as more aggressive cancers and difficult to treat^37^. Although this finding was not significant, it aligns well with existing literature. In a previous study, we found significantly elevated levels of miR-20a-5p in high grade tumors and in triple-negative breast cancer compared to other subtypes using miRNA microarray and quantitative polymerase chain reaction (qPCR)^11^. Additionally, c-myc, a well-established transcriptional regulator of the miR-17-92 cluster to which miR-20a-5p belongs, has been found to be upregulated in basal-like breast cancers^38,39^. In contrast, our results also indicate that a high cytoplasmic miR-20a-5p may be associated with lower odds of lymph node metastasis, contradicting the hypothesis that elevated cytoplasmic miR-20a-5p expression may be associated with a more aggressive tumor phenotype. However, none of these findings reached statistical significance at the 5% level, and further research with larger cohorts is warranted to better understand the underlying mechanisms and validate the observed associations.

In the cancer cell nucleus, miR-20a-5p expression was inversely associated with tumor size and lymph node metastasis, suggesting a potentially less aggressive tumor phenotype in women with high nuclear miR-20a-5p expression. This contrasts with findings from the cancer cell cytoplasm, which suggested a possibly more aggressive tumor phenotype. There is increasing evidence of miRNAs that localize and have specific functions in the nucleus^20–22,40^, and that transportation across the nuclear membrane can regulate miRNA storage and function^41^. Considering the observed discrepancy in tumor phenotype associations between compartments, it would be interesting to explore whether nuclear miR-20a-5p may have effects that differ from those of its cytoplasmic counterpart. Previous studies have already indicated that miRNAs may have different functions depending on tissue compartment and subcellular localization^21,22^. Nuclear miR-20a-5p could be sequestered or in an inactive form, although this possibility has not yet been investigated to our knowledge. The nuclear functions of miRNAs in general, and miR-20a-5p in particular, remain elusive and more research on tissue- and subcellular localization and function of miR-20a-5p is needed.

In terms of associations with demographic, anthropometric, lifestyle and reproductive factors, we observed a significantly lower median age at diagnosis among women with high stromal expression of miR-20a-5p and those with high cytoplasmic expression of miR-20a-5p. Furthermore, menopausal status at diagnosis showed associations with stromal and cytoplasmic miR-20a-5p expression, and with combined miR-20a-5p expression in cytoplasm and nucleus. Logistic regression analysis revealed increased odds of having high stromal and cytoplasmic miR-20a-5p expression in premenopausal women compared to postmenopausal women. This observation is biologically plausible, as premenopausal women have higher levels of circulating estradiol, and estradiol induces expression of transcriptional factors such as c-myc^42^ and E2F1^43^ which in turn regulate transcription of several miRNAs, including miR-20a-5p^44,45^. On a more general basis, miR-20a-5p, amongst others, has been identified as downregulated in cellular models of aging^46^. Moreover, we found that nuclear miR-20a-5p expression was associated with BMI. This observation is supported by recent studies, which indicate that the inflammation caused by adipose tissue may influence miRNA expression levels, and that miRNAs may be mediators of the effect of obesity on breast cancer development and progression^47,48^.

Our in vitro assays demonstrated that an increased expression of miR-20a-5p led to increased migration in all breast cancer cell lines and increased invasiveness in two out of three cell lines compared to controls. These results are in accordance with findings from cancer cell cytoplasm: cytoplasmic expression of miR-20a-5p in cancer cells was associated with tumor grade, and tumors with high compared to low cytoplasmic miR-20a-5p showed a trend towards higher odds of a basal-like subtype and high Ki67, potentially indicating a more aggressive tumor type. Our findings also support those of Bai et al., who reported that overexpression of miR-20a-5p in TNBC cells led to increased migration and invasion in vitro^16^. Similarly, Guo et al. found that miR-20a-5p promoted migration and invasion in MDA‐MB‐231 cells^15^. In contrast, Zhao et al. reported a reduction in invasive capabilities following miR-20a-5p transfection^17^. Variations in assay techniques and cell culture conditions may contribute to the discrepancies between study results.

There was no increase in proliferation following miR-20a-5p transfection in vitro*,* seemingly contradicting our finding from cancer cell cytoplasm where high cytoplasmic expression was associated with a non-significant increase in odds of high Ki67.

Overall, the cell studies support a possible association between high miR-20a-5p expression and a more aggressive disease course.

### Strengths

By combining data obtained within the natural tissue context with long follow-up time, epidemiological and clinicopathological data, and functional in vitro experiments, we comprehensively evaluate and integrate several aspects of miR-20a-5p’s expression and function in breast cancer.

One of the major strengths of our study lies in the information about miRNA localization obtained by ISH. The main techniques currently used for miRNA quantification are qPCR, microarray analysis, next-generation sequencing, Northern blotting, and isothermal amplification^24^, none of which provide information on the subcellular localization of miRNAs. To our knowledge, there are no previous studies that have evaluated the expression of miR-20a-5p in both tumor stromal fibroblasts, cancer cell cytoplasm and cancer cell nuclei and examined their associations with the clinical endpoints relapse, lymph node metastasis and distant metastasis, as well as other clinicopathological, reproductive and lifestyle factors. By identifying the precise locations of miRNAs, we gain valuable insights into their potential functional roles. Different subcellular locations could indicate distinct regulatory mechanisms and target interactions. The canonical understanding is that miRNAs carry out their function in the cytoplasm by targeting mRNAs to inhibit their translation or promote their degradation post-transcriptionally. However, we observed nuclear expression of mature miR-20a-5p, indicating a possible role in transcriptional regulation or other nuclear processes. Furthermore, identifying the precise locations of miRNAs may facilitate biomarker development, as miRNA expression patterns within specific tissue- and subcellular compartments can serve as diagnostic or prognostic indicators. Lastly, understanding the delivery sites of miRNAs is a prerequisite for the development of effective anti-miRNA therapeutic strategies^49^. Importantly, we observed a high inter-observer agreement in this evaluation, underscoring the reliability of our assessment methodology and enhancing the credibility of the reported findings.

Other strengths include the long follow-up time, and the employment of restricted cubic splines to allow for flexible modeling of non-linear associations. By using this statistical method, we were able to visualize the complex relationship between stromal miR-20a-5p expression and relapse.

### Limitations

Lack of reproducibility is a concern due to the semiquantitative scoring method and the absence of an established biologically relevant cut-off value. The subjective nature of scoring and the potential interobserver variability can affect the accuracy and reproducibility of our findings. Additionally, using small tissue cores to evaluate large tumors may have limitations due to tumor tissue heterogeneity.

Although surrogate markers are convenient and accessible indicators of molecular characteristics, they do not always accurately reflect the true molecular subtype, as determined by advanced gene expression analysis^50–52^. Thus, the use of surrogate markers to classify breast cancer into molecular subgroups may lead to misclassification. However, the surrogate markers used in our study reflect the prognostic and predictive markers commonly used in clinical practice to categorize and stratify tumors, underlining the translational relevance of our observations.

Furthermore, some of our analyses were underpowered due to a low number of participants in certain subgroups, specifically nulliparous women, women with obesity, those who had tumor relapse, those who had distant metastasis, and those with cancers of some subtypes. This may have limited our ability to detect significant associations. As such, our findings warrant further investigation in larger studies.

Additionally, while BMI and physical activity have been previously validated^53,54^, a misclassification of other lifestyle and reproductive factors of an unknown degree is likely present as the data on these factors were collected from self-administered questionnaires. There is also a time gap between the questionnaire data and clinical data collection for some participants, potentially leading to misclassification of the questionnaire responses. Moreover, some women (17.3%) completed the questionnaire after their breast cancer diagnosis, and we assumed that the reported information corresponded to that at the time of cancer diagnosis, which may not always be accurate.

Regarding the in vitro functional analyses, certain limitations arise from using cell lines originating from metastatic sites. Given that these cell lines have undergone metastasis, our observations predominantly correspond to the traits and actions of cells in the metastatic phase, rather than in the primary tumor. Further, our cell studies do not consider the tumor-stroma interplay and the role of the tumor microenvironment in tumor development and progression.

## Conclusions

While most of our results point towards an oncogenic role, some of our findings indicate that miR-20a-5p may have diverse effects based on tissue compartment and subcellular location.

The associations of stromal miR-20a-5p expression with relapse and high Ki67 expression, and of cytoplasmic miR-20a-5p with tumor grade, and possibly with high Ki67 expression and the basal-like subtype, suggest that miR-20a-5p may have oncogenic properties in the tumor microenvironment and in cancer cells. Conversely, the associations of nuclear miR-20a-5p expression with smaller tumors and with decreased odds of lymph node metastasis suggest a protective role. Thus, nuclear versus stromal and cytoplasmic miR-20a-5p expression may have opposing effects on breast cancer progression.

Taken together, our findings on miR-20a-5p, especially its differential expression in various tissue and subcellular compartments, contribute to the evolving landscape of precision medicine by identifying it as a potential biomarker for targeted therapies in breast cancer. However, establishing new biomarkers requires several phases^55^. The present study contributes to the first phase which is preclinical exploratory studies, and our findings may help prioritize future research to bridge the gap between current knowledge and the clinical utility of miR-20a-5p in breast cancer. In the broader context, harnessing the potential of new biomarkers requires both ethical, legal, and social considerations, and it requires prioritization from policymakers^2^.

Our findings warrant further research in larger studies on miR-20a-5p’s expression levels and functions within different tissue- and subcellular locations, and its potential clinical utility as a biomarker, treatment target or treatment tool in breast cancer.

## Supplementary Information

Supplementary Information.

## Data Availability

The datasets generated and analyzed during the current study are not publicly available due to the sensitivity of the data that has been collected from the CAMO cohort. However, data may be available upon request to NOWAC at nowac@uit.no and/or Aassociate Professor K.S.O.
