# Supplementary material for: Roles of miR-20a-5p in breast cancer based on the clinical and multi-omic (CAMO) cohort and in vitro studies
Source: Sci Rep. 2024 Oct 23;14:25022. doi: 10.1038/s41598-024-75557-0 (PMC11499649; doi:10.1038/s41598-024-75557-0)
Supplement: Supplementary file 1 — Supplementary Information. [file 41598_2024_75557_MOESM1_ESM.pdf]

List of abbreviations

BMI	Body mass index
CAF	Cancer associated fibroblast
CAMO	Clinical and Multi-omics cohort
CI	Confidence interval
DIG	Digoxigenin
ER	Estrogen receptor
FBS	Fetal bovine serum
FFPE	Formalin-fixed paraffin-embedded
HER2	Human epidermal growth factor receptor 2
HR	Hormone receptor
ISH	*In situ* hybridization
MiRNA	MicroRNA
MTT	3-(4,5-dimethylthiazol-2-yl)-2,5-diphenyltetrazolium bromide
NcRNA	Non-coding RNA
NOWAC	Norwegian Women and Cancer study
OR	Odds ratio
PBS	Phosphate buffered saline
PR	Progesterone receptor
qPCR	Quantitative polymerase chain reaction
REK	Regional Ethical Committee
TMA	Tissue microarray
TNBC	Triple negative breast cancer

Supplementary Table S1 Probes and reagents used in ISH

Probes and Reagents	Reference	Company
Hsa-miR-20a-5p. miRURY LNA detection probe	6110011-360	Exiqon
Scramble-miR. miRURY LNA detection control probe	99004-15	Exiqon
U6, hsammurno. miRURY LNA detection control probe	99002-15	Exiqon
microRNA ISH Bufferset (FFPE)	9000	Exiqon
RiboWash SSPE buffer RUO	5266262001	Roche
Discovery EZ Prep	5279755001	Roche
LCS (Liquid cover slip)	5264839001	Roche
Reaction buffer (10x)	5353955001	Roche
Discovery CC1 RUO	6414575001	Roche
Discovery Antibody block RUO	5268869001	Roche
Anti DIG AP Multimer	7256302001	Roche
ChromoMap Blue kit RUO	526661001	Roche
Red Stain II	5272017001	Roche
Ethanol 96%	20823.362	VWR, Avantor
Etnanol absolute	20821.296	VWR, Avantor
Xylene	28975.291	VWR, Avantor
Histokitt mounting medium	Assistant 1025/250	Sondheim/Rhoen

Supplementary Table S2 ISH protocol

	MiR-20a-5p	U6	Scramble miRNA
**Deparaffinization**	EZ Prep, 3x12 min, 68°C	EZ Prep, 3x12 min, 68°C	EZ Prep, 3x12 min, 68°C
**Heat retrieval**	Discovery CC1, 40 min, 95 ^{o}C	Discovery CC1, 40 min, 95 ^{o}C	Discovery CC1, 40 min, 95 ^{o}C
**Denaturation**	8 min, 90 ^{o}C	8 min, 90 ^{o}C	8 min, 90 ^{o}C
**Hybridization**<br>Concentration<br>Buffer	60 min, 40°C<br>50nM<br>microRNA ISH Bufferset	60 min, 55 ^{o}C<br>0.5nM<br>microRNA ISH Bufferset	60 min, 57 ^{o}C<br>10nM<br>microRNA ISH Bufferset
**Stringency wash**	RiboWash, 2x8 min, 40°C	RiboWash, 2x8 min, 55°C	RiboWash, 2x8 min, 57°C
**Blocking**	AB block, 16 min, 37°C	AB block, 16 min, 37°C	AB block, 16 min, 37°C
**Secondary multimer**	Anti-DIG-AP, 20 min, 37°C	Anti-DIG-AP, 20 min, 37°C	Anti-DIG-AP, 20 min, 37°C
**Detection**	Blue kit, 120 min, 37°C	Blue kit, 120 min, 37°C	Blue kit, 120 min, 37°C
**Counter stain**	Red II, 4 min, 37°C	Red II, 4 min, 37°C	Red II, 4 min, 37°C
**Used in multiple steps**<br>Coverslip solution<br>Washing buffer	 LCS (Liquid cover slip)<br>Reaction buffer (10x)	 LCS (Liquid cover slip)<br>Reaction buffer (10x)	 LCS (Liquid cover slip)<br>Reaction buffer (10x)

Supplementary figure S1 Proliferation

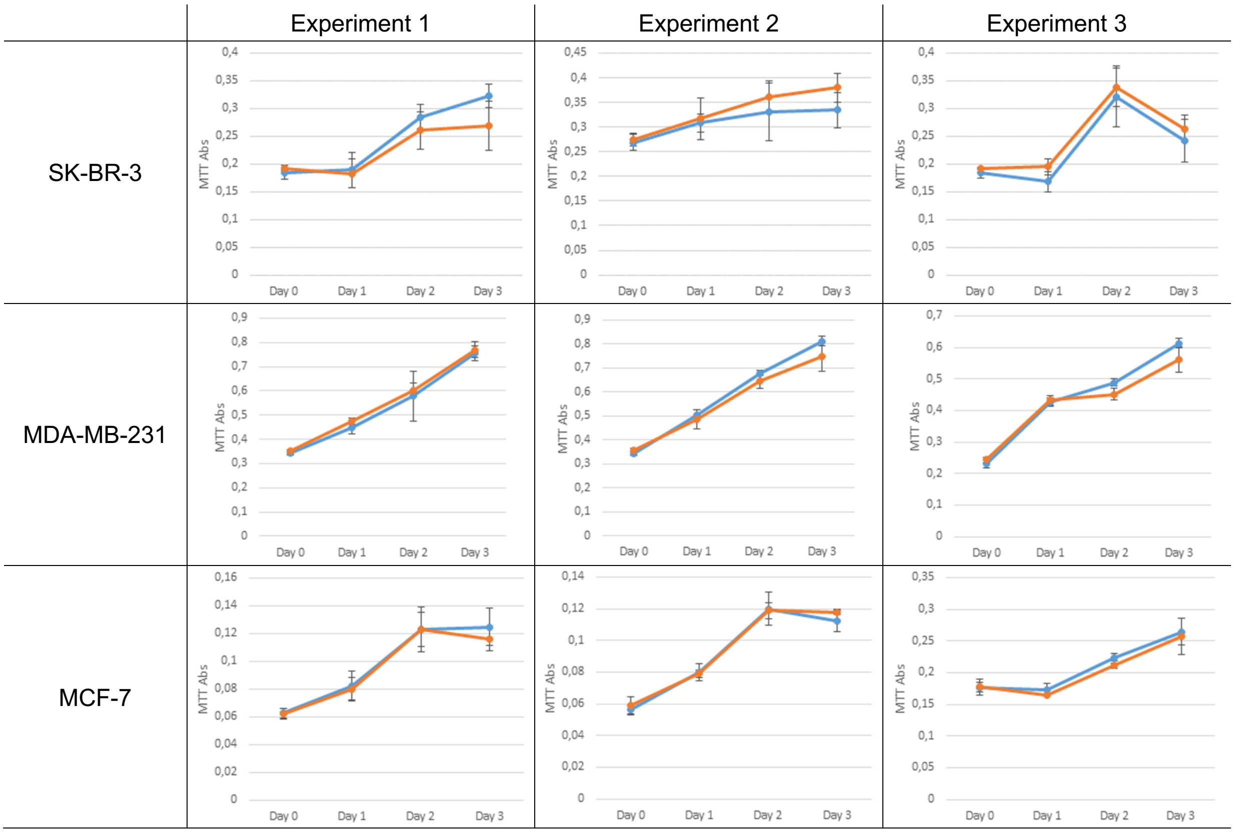

MTT assay to assess the effects of miR-20a-5p transfection on cell proliferation in the breast cancer cell lines SK-BR-3, MDA-MB-231, and MCF-7. Blue lines: cells transfected with miR-20a-5p mimic. Orange lines: negative control.
